# *Trypanosoma cruzi* in Persons without Serologic Evidence of Disease, Argentina

**DOI:** 10.3201/eid0912.030008

**Published:** 2003-12

**Authors:** Oscar A. Salomone, Ana L. Basquiera, Adela Sembaj, Ana M. Aguerri, María E. Reyes, Mirtha Omelianuk, Ruth A. Fernández, Julio Enders, Atilio Palma, José Moreno Barral, Roberto J. Madoery

**Affiliations:** * Hospital Privado Centro Médico de Córdoba, Córdoba, Argentina; † Universidad Nacional de Córdoba, Córdoba, Argentina; ‡ Ministerio de Salud de Córdoba, Córdoba, Argentina

**Keywords:** Chagas disease, polymerase chain reaction, *Trypanosoma cruzi*, diagnosis

## Abstract

Current diagnosis of chronic Chagas disease relies on serologic detection of specific immunoglobulin G against *Trypanosoma cruzi*. However, the presence of parasites detected by polymerase chain reaction (PCR) in patients without positive conventional serologic testing has been observed. We determined the prevalence and clinical characteristics of persons with seronegative results for *T. cruzi* DNA detected by PCR in a population at high risk for chronic American trypanosomiasis. We studied a total of 194 persons from two different populations: 110 patients were recruited from an urban cardiology clinic, and 84 persons were nonselected citizens from a highly disease-endemic area. Eighty (41%) of persons had negative serologic findings; 12 (15%) had a positive PCR. Three patients with negative serologic findings and positive PCR results had clinical signs and symptoms that suggested Chagas cardiomyopathy. This finding challenges the current recommendations for Chagas disease diagnosis, therapy, and blood transfusion policies.

American trypanosomiasis or Chagas disease is usually asymptomatic; for this reason, its diagnosis is mainly based on laboratory tests. During the indeterminate and chronic clinical periods, detection of immunoglobulin (Ig) G against *Trypanosoma cruzi* by more than two different serologic tests is the standard for diagnosis (*1*). Moreover, serodiagnosis is used for epidemiologic surveillance, to evaluate efficacy of therapy, and for routine testing in blood banks.

Conversely, direct identification of *T. cruzi* is the main tool for diagnosis during the acute phase of Chagas disease. During the other phases of the disease, detection of the parasites is rare because of low levels of parasitemia. However, the development of polymerase chain reaction (PCR) has allowed detection of *T. cruzi* in a higher number of patients with chronic disease. In this stage, the prevalence of circulating parasites varies from 21% to 100% by using PCR, and this variability may be associated with episodes of reinfection (*2*–*4*). Previous reports have focused on the high sensitivity of PCR test when compared to serologic findings, xenodiagnosis, or blood culture. Nonetheless, in some of these investigations a discordant finding has been observed; parasitemias have been detected by PCR from serum samples of seronegative persons (*5*–*9*). Although the parasite has been directly observed in blood of seronegative patients (*5*), this problem has been largely ignored in the clinical setting. Parasitemias in patients with negative serologic findings could represent a sanitary problem since most diagnostic and therapeutic recommendations rely on a serologic test.

We conducted a cross-sectional study in two populations at high risk for Chagas disease to evaluate the prevalence of positive *T. cruzi* PCR results in seronegative persons. We describe the results of that study as well as the clinical characteristics of a subgroup of patients.

## Patients and Methods

### Population and Protocol Study

We studied 194 persons from two populations. We included an urban population of 110 consecutive patients who attended the Cardiology Clinic of the Hospital Privado de Córdoba, Argentina, with epidemiologic or clinical suspicion of Chagas disease. All the patients were permanent residents of the city of Córdoba during the last 10 years. Córdoba is considered a low Chagas-endemic area. The other group consisted of 84 persons from La Posta, a small rural village of 384 persons located in a northern rural area of the province of Córdoba. This area is highly endemic for Chagas disease. All residents of this area >14 years of age were invited to participate in the study through informative workshops conducted by specially trained sanitary agents. The study protocol was designed according to Helsinki’s Declaration, and informed consent was obtained for all patients.

All patients completed an epidemiologic and clinical questionnaire and had a physical examination. Also, both urban and rural participants had a 12-lead electrocardiogram and a transthoracic echocardiogram.

### Serologic Tests

Three serologic assays for all case-patients were performed to detect chronic *T. cruzi* infection: indirect immunofluorescence assay (IFA, positive >1:32 dilution; Biocientífica, Buenos Aires, Argentina), hemagglutination inhibition assay (positive >1:28 dilution, Biochagas, Biocientífica, Buenos Aires, Argentina), and enzyme-linked immunosorbent assay (ELISA, Abbott Labs, Abbott Park, Illinois). Chronic Chagas disease was defined as the presence of >2 positive serologic determinations (*1*). Also, anti-cruzipain antibodies were detected by ELISA as previously described (*10*,*11*).

### PCR for Identification of *Trypanosoma cruzi*

Peripheral blood samples were drawn from each study participant for PCR detection of *T. cruzi,* as previously described (*12*,*13*). Four milliliters of blood was transferred to guanidine-EDTA containing tubes until DNA extraction. We collected 600 mL of blood to separate DNA by using conventional technique of fenol: chloroform: isoamyl alcohol and then ethanol precipitation. Finally, the solution was suspended in free-endonuclease sterile water. DNA amplification was carried out in 50 mL of a mixture containing 10 mM 10 mM Tris (pH 8.3), 50 mM KCl, 1.5 mM MgCl2, 0.2 mM of each deoxinucleoside triphosphate, 1.25 U Taq polymerase (Perkin Elmer Cetus Corp, Norwalk, CT), and 1 mM of each primer. We amplified a sequence of 220 bp, which corresponds to a family of E13 genes with high repetition in the genome of *T. cruzi*; the sequence of the primers used was: O1, 5′-TGGCTTGGAGGAGTTATTGT-3′; O2, 5′-AGGAGTGACGGTTGATCAGT-3′ (*12*). The reaction was initiated with 10 min of denaturalization at 94°C and 30 cycles of amplification, each consisting of 1 min at 94°C, 1 min at 55°C, and 2 min at 63°C in a Perkin-Elmer-Cetus terminal cycler. We analyzed the PCR product in a 1.6% agarose gel. In all samples, DNA from cultivated *T. cruzi* epimastigotes of Tulahuen strain was used as positive control. Negative control consisted of a specimen without DNA. Also, 330-bp fragment of the β-actin gene (Promega, Madison, WI) was amplified with the same procedure as E13 fragment to check DNA quality and to show amplification inhibitors.

### Statistical Analysis

Data are presented as mean ±SD or as number and percentage of cases. We used chi-square test to compare categorical variables between groups. A value of p < 0.05 was considered significant.

## Results

Characteristics of both urban and rural populations are shown in Table 1. Results of serologic testing for 76 (69%) and 38 (44%) persons from urban and rural populations, respectively, were positive for *T. cruzi* infection. Globally, 80 (41%) persons did not fulfill criteria of serologic diagnosis of Chagas disease (in all cases, IFA test was negative). In eight of nine rural seronegative patients, anti-cruzipain antibodies were investigated with negative results.

**Table 1:** Demographic and laboratory characteristics of study participants^a^

Variable	Total (n = 194)	Urban population (n = 110)	Rural population (n = 84)
Age, mean ± SD (y)	52 ±14	56 ±14	48 ±15
Male (%)	36	37	33
Negative serologic finding, n (%)	80 (41)	34 (31)	46 (54)
Positive PCR assay, n (%)	34 (17)	14 (13)	20 (24)
Positive PCR assay and negative serologic findings, n (%)	12 (6)	3 (9)	9 (20)

^a^PCR, polymerase chain reaction.

*T. cruzi* was detected by PCR amplification of a nuclear DNA fragment by using the O1/O2 primers (see Materials and Methods). This reaction has been previously demonstrated to be highly specific to detect *T. cruzi* in blood samples (*12*). Parasitemia by PCR assay was detected in 34 (17%) of 194 persons and was more frequently found in rural than in urban populations (20 and 14 positive persons, respectively; p = 0.05) (Figure). When only the seronegative population was considered, PCR was positive in 12 (15%) persons (3 and 9 from urban and rural population, respectively; p = 0.36). Clinical characteristics of these patients are shown in Table 2. Only one patient (from the urban group) had a previous positive Machado Guerreiro test. Two of three urban patients were born in a highly disease-endemic area. Disease in all of these urban patients was controlled a year after recruitment, and subsequent serologic testing was negative. Of the rural case-patients (born and living in La Posta), none reported previous positive serologic findings. EKG and echocardiogram were performed for four patients from a rural area (Table 2).

**Figure:**
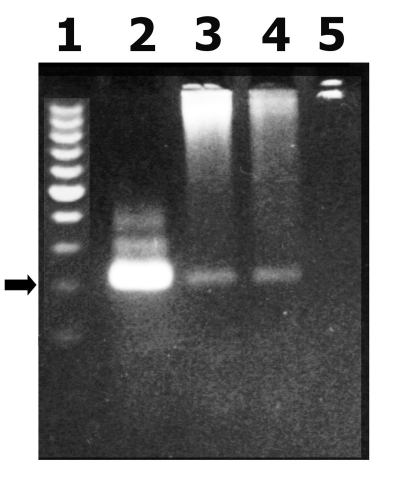
Gel electrophoresis analysis of a polymerase chain reaction (PCR) product corresponding to a highly repetitive 220-bp *Trypanosoma cruzi* nuclear fragment. 1: molecular weight standards, 2: *T . cruzi* nuclear 220-bp PCR product, 3 and 4: PCR product from patients blood, 5: PCR negative control (arrows correspond to 220 bp).

**Table 2:** Epidemiologic and clinical characteristics of 12 patients with negative serologic findings and positive PCR for *T. cruzi* in blood^a^

Patient	Age (y)	Sex	Previous positive serologic test	EKG	Echocardiogram	
					LVDd (mm)	LVEF (%)
Urban 1	56	F	Present	Normal	44	64
Urban 2	66	F	Absent	RBBB	42	65
Urban 3	58	M	Absent	RBBB + LAFB	45	60
Rural 1	35	F	Absent	ND	ND	ND
Rural 2	17	F	Absent	ND	ND	ND
Rural 3	15	F	Absent	ND	ND	ND
Rural 4	47	F	Absent	Normal	45	50
Rural 5	59	M	Absent	ND	ND	ND
Rural 6	22	F	Absent	ND	ND	ND
Rural 7	24	M	Absent	IRBBB	50	45
Rural 8	68	M	Absent	Normal	39	56
Rural 9	43	F	Absent	Normal	46	64

^a^PCR, polymerase chain reaction; F, female; M, male; EKG, electrocardiogram; RBBB, right bundle branch block; IRBBB, incomplete right bundle branch block; LAFB, left anterior fascicular block; LVDd, left ventricular diameter in diastole; LVEF, left ventricular ejection fraction; ND, not determinate.

## Discussion

When *T. cruzi* infects a mammal, several immunologic reactions occur that eliminate the parasite. First, a cellular immune response attempts to isolate the microorganism and avoid its wide spread. Simultaneously, a humoral response occurs, with IgM antibodies first and IgG antibodies 2–3 weeks later. However, because of the lack of efficacy of these mechanisms, the parasite persists in low-density tissues and in turn, triggers an inflammatory response, resulting in tissue damage during the chronic period of the disease (*14*). Parasites are rarely isolated from blood or tissue from chronically infected patients, and the diagnosis is based on serologic analysis.

In our study, we observed that persons with positive *T. cruzi* in blood and negative serologic findings could be detected in a population with high epidemiologic risk. This observation has been previously reported in Wincker et al. (*5*), who studied PCR technique using serum samples from 45 Bolivian children. They found two positive PCR results in 17 seronegative children, and in one of them, parasites were seen on direct blood examination. These authors also reported a patient with the same infectious condition in 268 children with high epidemiologic risk for Chagas disease (*6*). In Brazil, Avila et al. (*7*) observed three discordant cases, one of which had typical findings of myocardial damage. Similarly, Castro et al. (*9*) detected 3 persons with positive PCR results among 9 seronegative controls, and Gomez et al. (*8*), reported 10 positive PCR results in seronegative patients of 110 residents of a highly disease-endemic region.

Several arguments have been proposed to explain this controversial situation. Recent infection that has not yet been recognized by the immune system of persons highly exposed to vectorial infections is one possible explanation. However, acute infection is not a frequent event in our study population because of age and because urban population is infrequently exposed to vectorial reinfection. Alternatively, one could speculate that positive samples could have been contaminated with DNA, but this theory has been disregarded by many authors (*8*,*9*). We repeated serologic and PCR assays three times for each patient with two different operators, and we obtained the same results. Finally, *T. cruzi* may chronically infect some patients but a humoral response may not develop or be detected by conventional serologic testing. Addressing this point, Castro et al. (*9*) observed that 80% of seronegative but positive PCR patients had lytic antibodies against *T. cruzi* by a complement-mediated lysis test (CoML). Similarly, Leguizamón et al. (*15*) have reported patients who were seropositive for Chagas disease only by inhibition transamidase assay but negative with conventional serologic testing. To test this hypothesis, we searched for anti-cruzipain antibodies in eight rural patients, but all of them were negative.

Otherwise, independently of its cause, considering the clinical and diagnostic consequences of this phenomenon is necessary. In our study, at least 3 of the 12 patients with high epidemiologic risk for Chagas disease had signs of cardiac compromise. The consensus is that the detection of DNA constitutes real proof of parasites. DNA detected in blood is originated from extracellular parasites recently liberated or destroyed. According to this theory, Tarleton and Zhang observed that after injection of high doses of kinetoplastic DNA (kDNA) of *T. cruzi* in muscle, kDNA is detected in blood 2 days later (*16*). However, since the parasite is infective as tripomastigotes but not as a portion of DNA, we cannot be sure that DNA detected by PCR in blood is a reliable surrogate of infecting *T. cruzi* forms. One alternative is the possibility that fragments of amastigotes (the tissue-infecting forms) reach the bloodstream after interacting with the immune system. Even though this consideration may be found relevant in the design of transfusion policies, no reliable information currently exists, and the potential for these persons to transmit the disease is still uncertain.

Comparing our results with a population without any risk for Chagas disease to determine the predictive value of PCR among seronegative persons would be interesting. However, the accuracy of PCR has been studied in depth (*2*–*4*,*12*,*17*). While previous reports of PCR in Chagas disease used a sequence of kDNA of *T. cruzi* to detect the parasite, we used nuclear DNA that has been also validated (*12*,*13*). Currently, we have not carried out a systematic comparison of the PCR sensitivity for different *T. cruzi* sequences. We considered determining which of the different PCR systems cited in the literature is the more sensitive and accurate for detection of parasitemia in blood specimens of patients with chronic Chagas disease.

In summary, we found a prevalence of 15% of *T. cruzi* DNA for American trypanosomiasis in a seronegative population living in Chagas-endemic regions. We also observed that some of these persons had cardiac abnormalities suggestive of Chagas cardiomyopathy. Experts should consider these finding when making diagnostic, therapeutic, and transfusion recommendations.
